# Amlexanox attenuates experimental autoimmune encephalomyelitis by inhibiting dendritic cell maturation and reprogramming effector and regulatory T cell responses

**DOI:** 10.1186/s12974-019-1438-z

**Published:** 2019-03-01

**Authors:** Mo-Yuan Quan, Xiu-Juan Song, Hui-Jia Liu, Xiao-Hong Deng, Hui-Qing Hou, Li-Ping Chen, Tian-Zhao Ma, Xu Han, Xin-Xin He, Zhen Jia, Li Guo

**Affiliations:** Department of Neurology, The Second Hospital of Hebei Medical University, Key Laboratory of Hebei Neurology, No. 215 Heping Road, Shijiazhuang, 050000 Hebei China

**Keywords:** Amlexanox, Dendritic cells, TBK1, IRF3, AKT, Experimental autoimmune encephalomyelitis

## Abstract

**Background:**

Amlexanox (ALX), a TBK1 inhibitor, can modulate immune responses and has anti-inflammatory properties. To investigate its role in regulating the progression of experimental autoimmune encephalomyelitis (EAE), we studied the effect of ALX on the maturation of dendritic cells (DCs) and the responses of effector and regulatory T cells (Tregs).

**Methods:**

In vitro, bone marrow-derived DCs (BMDCs) were cultured and treated with ALX. Their proliferation, maturation, and their stimulatory function to induce T cells responses were detected. In vivo, the development of EAE from different groups was recorded. At the peak stage of disease, HE, LFB, and electronic microscope (EM) were used to evaluate inflammation and demyelination. Maturation of splenic DC and Th1/Th17/Treg response in the CNS and peripheral were also detected. To further explore the mechanism underlying the action of ALX in DC maturation, the activation of TBK1, IRF3, and AKT was analyzed.

**Results:**

Our data indicated that ALX significantly inhibited the proliferation and maturation of BMDCs, characterized by the reduced MHCII, a co-stimulatory molecule, IL12, and IL-23 expression, along with morphological alterations. Co-culture of ALX-treated BMDCs inhibited allogeneic T cell proliferation and MOG-specific T cell response. In EAE mice, ALX significantly attenuated the EAE development by decreasing inflammatory infiltration and demyelination in the spinal cords, accompanied by reduced frequency of splenic pathogenic Th1 and Th17 cells and increased Tregs. Moreover, ALX treatment decreased Th1 and Th17 cytokines, but increased Treg cytokines in the CNS and spleen. Notably, ALX treatment reduced the frequency and expression of CD80 and CD86 on splenic DCs and lowered IL-12 and IL-23 secretion, further supporting an impaired maturation of splenic DCs. In addition, ALX potently reduced the phosphorylation of IRF3 and AKT in BMDC and splenic DCs, both of which are substrates of TBK1 and associated with DC maturation.

**Conclusions:**

ALX, a TBK1 inhibitor, mitigated EAE development by inhibiting DC maturation and subsequent pathogenic Th1 and Th17 responses while increasing Treg responses through attenuating the TBK1/AKT and TBK1/IRF3 signaling.

## Background

Amlexanox (ALX) has anti-inflammatory and anti-allergic properties and has been used for the treatment of several diseases, such as aphthous ulcers and asthma [1, 2]. Recent studies have shown that ALX, as a specific inhibitor of TANK-binding kinase 1(TBK1)/IkB kinase ε (IKKε), can suppress chronic inflammation in type II diabetes [3], non-alcoholic fatty liver [4], and obesity-related metabolic dysfunction [5]. However, whether ALX can modulate the pathogenic process of multiple sclerosis (MS) has been rarely reported and poorly understood.

MS is a chronic inflammatory autoimmune disease characterized by infiltration of myelin-specific pathogenic T cells into the central nervous system (CNS), where they secrete inflammatory cytokines, leading to demyelination and neuronal injury [6, 7]. Previous studies have demonstrated that pathogenic Th1 and Th17 cells are crucial for the pathogenesis of MS and experimental autoimmune encephalomyelitis (EAE), the murine model of MS [8, 9]. These antigen-specific Th1 and Th17 cells secrete pro-inflammatory interferon-γ (IFN-γ) and interleukin (IL)-17, respectively, during the process of EAE and MS [10, 11]. IFN-γ can activate dendritic cells (DCs), monocytes, macrophages, and microglial cells, and IL-17 can stimulate chemokine production to attract neutrophils into the lesions to enhance inflammation [12].

Regulatory T cells (Tregs) are believed to maintain immunological tolerance in MS by preventing or downregulating inflammation [12]. FOXP3, a specific transcription factor of CD4^+^CD25^+^ Treg cells, is crucial for their suppressive function [13]. IL-10-secreting T regulatory type 1 (Tr1) cells can suppress antigen-specific effector T cell responses via a cytokine-dependent mechanism [14]. Naïve CD4^+^ T cells can be activated and differentiate into effector and regulatory T cells, which heavily depends on various stimulations from antigen presenting cells (APCs) [15, 16].

DCs are the most potent APCs and are essential for inducing naïve T cell activation and subsequent functional differentiation [17, 18]. The activated and mature DCs express high levels of MHC II and co-stimulatory molecules, such as CD80 and CD86, and secrete large amounts of pro-inflammatory cytokines. These pro-inflammatory cytokines, including IL-12, IL-6, and IL-23, can drive Th1 and Th17 cell differentiation and functional development, leading to inflammation and autoimmunity [18, 19]. While immature DCs have potent capacity to capture antigens, the low expression of co-stimulatory molecules and cytokines may induce Tregs and peripheral tolerance [20, 21]. Therefore, controlling the extent of DC activation and maturation is crucial to regulating immune homeostasis and the balance between T cell immunity and tolerance. However, there is very limited information available on whether ALX can modulate the maturation of DCs in an EAE environment.

TBK1 and its homolog IκB kinase epsilon (IKKε) are the non-canonical IKKs [22] and involved in the pathogenic process of neuroinflammation [23, 24] and autoimmune responses [25, 26]. In MS patients, TBK1 expression is upregulated in peripheral blood mononuclear cells (PBMCs), indicating its vital role in the occurrence and development of MS [27, 28]. A recent study through ingenuity pathway analysis between semi-mature and fully mature DCs also reveals that TBK1 expression is upregulated in induced-matured DCs, suggesting its critical role in regulating DC maturation [29]. TBK1 can also phosphorylate and activate IRF-3, which acts as a promoter of DC maturation [30, 31]. Downregulating IRF-3 in DCs through small interfering RNA, or drugs such as zoledronic acid, results in both phenotypic and functional immaturity [31, 32]. In addition, recent studies have indicated that activation of the TBK1/AKT signaling is necessary for TLR-driven glycolysis during DC activation, and inhibition of AKT can impede the activation and maturation of bone marrow DCs (BMDCs) [33]. Accordingly, we hypothesized that treatment with ALX, an inhibitor of TBK1, could suppress DC maturation and subsequently reprogram the balance between effector and regulatory T cells, attenuating EAE development.

## Methods

### Animals

Female C57BL/6 and BALB/c mice, 6–8 weeks old and weighing 18–20 g, were from Vital River Laboratories (Beijing, China). The mice were housed in a specific pathogen-free animal facility with a 12-h dark/light cycle and free access to food and water. The experiments were performed in accordance with the guidelines for animal care, and the experimental protocols were approved by the Institutional Animal Care and Use Committee of Hebei Medical University.

### Induction, treatment, and evaluation of EAE

C57BL/6 mice were immunized subcutaneously in the back region with myelin oligodendrocyte glycoprotein peptide MOG35-55 (250 μg per mouse, Lysine Bio-system, Xian, China) in 50% complete Freund’s adjuvant (CFA, Sigma, St. Louis, MO, USA), containing 4 mg/ml heat-inactivated *Mycobacterium tuberculosis* H37Ra (Difco Laboratories, Detroit, MI, USA). On day 0 and 2, the mice were injected intraperitoneally with pertussis toxin (500 ng per mouse, Alexis, San Diego, CA, USA). The mice were randomized and administrated orally with vehicle or ALX at 50 mg/kg twice daily beginning on the immunization day.

The mice were weighed and examined daily up to 29 days post-immunization. The disease severity was scored in a blinded manner as the following: 0, no obvious changes in motor functions; 1.0, limp tail; 2.0, limp tail and wobbly gait; 3.0, bilateral hind limb paralysis; 4.0, complete hind limb and partial forelimb paralysis; and 5.0, death [34].

### BMDC viability and proliferation assay

The bone marrow cells were freshly isolated from tibia and femur bones of C57BL/6 mice, and cultured in Petri dishes at 37 °C 5% CO_2_ in RPMI 1640 medium supplemented with 10% FBS, 1 mM sodium pyruvate, 2 mM L-glutamine, 100 μg/ml kanamycin, and 20 ng/ml GM-CSF (PeproTech, Rocky Hill, USA) to generate BMDCs [35]. After 8-day culture, BMDCs were treated with ALX at different concentration (2 to 200 μM) for 12 h. Their apoptosis and viability were analyzed using Annexin V-PE and 7AAD Apoptosis Detection Kit I (US Everbright) and Cell Counting Kit-8 (CCK-8) assay kit (US Everbright, Suzhou, China), respectively. A portion of BMDCs was stimulated with LPS (1 μg/ml) in the presence or absence of different concentrations (2 to 50 μM) of ALX for 48 h to induce DC maturation and activation [32]. The cell proliferation was determined using the CCK8 assay kit (US Everbright), according to the manufacturer’s instruction [16, 36].

### Transmission electron microscopy and scanning electron microscopy

BMDCs (10^6^/ml) were harvested on day 8 post-culture and stimulated with LPS (1 μg/ml) in the presence or absence of ALX (10 μM) for 2 days. After being washed twice with PBS, the cells were fixed with 2.5% glutaraldehyde and post-fixation in 1% osmic acid for 2 h. The specimens were dehydrated in acetone and embedded in Epon 812. The ultrathin sections (70 nm) were examined in a TEM (JEOL JEM-1230EX).

The harvested BMDCs (10^6^/ml) were stimulated with LPS (1 μg/ml) in the presence or absence of ALX (10 μM) for 2 days on pre-coated coverslips and fixed in 3% glutaraldehyde at 4 °C for 90 min, followed by post-fixation in 1% osmic acid for 20 min. The samples were dehydrated in ethanol for 10 min. Following cold sputter coated with gold, all samples were observed in a SEM (JEOL JSM-5600LV).

On days 24–26 post-immunization (the peak stage of EAE), some mice (*n* = 6 per group) were sacrificed. The white matter of the lumbar spinal cords of individual mice was dissected, cut into small blocks, and pre-fixed with 4% (*v*/*v*) glutaraldehyde. The blocks were washed and post-fixed in 1% (*w*/*v*) buffered osmium tetroxide for 1 h and dehydrated with graded acetone; the blocks were embedded in epoxy resin Epon 812. The ultrathin sections (70 nm) were stained with uranyl acetate and lead citrate. The intensity of demyelination was examined by TEM in a JEM-1230 electron microscope (JEOL, Japan).

### Antigen-specific T cell responses

After 7 day culture, BMDCs were loaded with MOG (10 μg/ml) [37] for 24 h and stimulated with LPS (1 μg/ml) in the presence or absence of ALX (2 or 10 μM) for 48 h. In addition, some of BMDCs were pretreated with mitomycin C (30 μg/ml, Sangon Biotech) for 30 min. Splenic CD4+ T cells were isolated from the MOG35-55-immunized C57BL/6 mice by negative selection. The purified CD4+ T cells were co-cultured with different groups of DCs at a ratio of 5:1, respectively. The cells were cultured for 4 days, and the suspended T cells were stained with fluorescent-labeled anti-CD25 and anti-CD69 (eBioscience, San Diego, CA, USA) [38, 39] to measure T cell activation; the cultured supernatants were collected for measuring the levels of IL-2 and IFNγ using specific kits (USCN LIFE). The T cell proliferation was determined by CCK-8 assay (US Everbright).

### Mixed lymphocyte reaction (MLR)

Splenic mononuclear cells were isolated from BALB/c mice, and CD4^+^ T cells were purified by negative selection using magnetic beads and the specific kit, as described by the manufacturer’s instruction (Miltenyi Biotech, Auburn, CA, USA). BMDCs were stimulated with LPS alone or together with ALX at 2 or 10 μM for 48 h; these cells were purified by positive selection using magnetic beads (Miltenyi Biotech) and treated with mitomycin C (30 mg/L, Sangon Biotech, Shanghai, China) for 30 min before harvesting, and washed with PBS twice to terminate the influence of drug on T cell proliferation. The different groups of BMDCs were co-cultured in triplicate with 1 × 10^5^ CD4^+^ T cells at a ratio (DC:T) of 1:5, 1:10, or 1:20, respectively in 96-well plates for 3 days. The CD4^+^ T cells alone served as the control. The proliferation in individual groups of T cells was determined by CCK-8 assay. The proliferation index (PI) was calculated as PI = (co-culture well OD value-medium OD value)/(T cell well OD value-medium OD value) [40].

### Splenic CD4+ T cells and DC isolation

Splenic CD4+ T cells were isolated by negative depletion (Miltenyi Biotech) from the mice at 25 days post-immunization with MOG35-55 in CFA. Splenic DCs were purified using anti-CD11c-conjugated magnetic beads (Miltenyi Biotech) from the mice at 15 days post-immunization with MOG35-55 in CFA. Absolute numbers of splenic mononuclear cells, DCs, and CD4+ T cells were calculated.

### Flow cytometry

The harvested BMDC (2 × 10^6^ /well) were stimulated in triplicate with LPS (1 μg/mL), alone or together with ALX at 2 or 10 μM in 6-well plates for 2 days. The cells were stained with PE-anti-CD11c, Percp-cy5.5-anti-MHC-II, FITC-anti-CD80 or FITC-anti-CD86, or isotype controls (eBioscience), washed, and analyzed by flow cytometry.

On days 24–26 post-immunization, six mice from each group were sacrificed, and their splenic mononuclear cells were isolated. The cells were stimulated with Cell Stimulation Cocktail (PMA + Ionomycin, eBioscience) for 5 h. The cells were stained with FITC-anti-CD4 (eBioscience), fixed, and permeabilized. After being washed, the cells were stained intracellularly with PE-anti-IFN-γ or PE-anti-IL-17 (eBioscience). The frequency of Th1 (CD4^+^IFN-γ^+^) and Th17 (CD4^+^Th17^+^) cells in total CD4^+^ T cells was determined by flow cytometry.

On days 15 post-immunization (the early stage of EAE), splenic mononuclear cells were isolated from the vehicle or ALX-treated EAE mice. The cells were stimulated with 20 μg/ml of MOG35-55 for 48 h and stained with PE-anti-CD11c, Percp-cy5.5-anti-MHC-II, FITC-anti-CD80 or FITC-anti-CD86, or isotype controls (eBioscience), and their expression levels were characterized by flow cytometry.

### Western blot analysis

After being cultured for 10 days, the different groups of BMDCs were harvested and lysed in RIPA buffer containing 1% phosphatase inhibitor cocktail, and 1 mM PMSF. The protein concentrations of individual samples were determined by BCA protein reagent kit (Novagen, Madison, WI, USA). The cell lysates (30 μg/lane) were separated by sodium dodecyl sulfate-polyacrylamide gel electrophoresis (SDS-PAGE) on 12% gels and transferred onto polyvinylidene difluoride (PVDF) membrane. The membranes were blocked with 5% BSA in TBST and incubated with rabbit monoclonal antibodies against β-actin, TBK1, phosphorylated TBK1 (Ser 172), AKT, phosphorylated AKT (Thr 308 and Ser 473), IRF3, phosphorylated IRF3 (Ser 396) (1:1000, Cell Signaling Technology, Beverly, MA, USA). The bound antibodies were detected with horseradish peroxidase-conjugated goat anti-rabbit immunoglobulin G (Rockland, Gilbertsville, USA) and visualized using the enhanced chemiluminescent reagents. The data were analyzed by an imaging densitometer (LI-COR Bioscience, Lincoln, NE, USA).

### Histopathology and immunofluorescence

On days 24–26 post-immunization, some mice were sacrificed (*n* = 4 per group) and their spinal cords were dissected. The spinal cord tissues were fixed in 10% formalin in PBS and paraffin-embedded. The spinal cord sections (5 μm) were stained with hematoxylin and eosin (H&E) and Luxol Fast Blue (LFB) to evaluate inflammation and demyelination. The degrees of inflammation (HE scores) were calculated as 0, normal; 1, cellular infiltrates partially around meninges and blood vessels; 2, 1–10 lymphocyte infiltrates in the parenchyma; 3, 11–100 lymphocyte infiltrates in the spinal cord; and 4, over 100 lymphocyte infiltrates in the spinal cord [41]. The severity of demyelination (LFB scores) was analyzed as 0, normal; 1, myelin sheath injuries in rare areas; 2, a few areas of demyelination; 3, confluent perivascular or subpial demyelination; 4, massive demyelination involving one half of the spinal cord; and 5, extensive demyelination involving the whole cord [42].

The spinal cord tissue sections (4 μm) from individual groups of mice were deparaffinized, rehydrated, and subjected to antigen retrieval. The sections were treated with 3% BSA in PBS and incubated with anti-F4/80, anti-IFN-γ, anti-IL17A, or anti-IL-10 (1:100, Sevicebio, Wuhan, China). After being washed, the sections were covered with secondary antibody and co-stained with DAPI. The fluorescent signals were observed under a fluorescent microscope to evaluate in the extensity of macrophages, Th1, Th17, and Tr1 infiltrates, respectively. In addition, the spleen tissues were dissected from individual mice on day 15 post-induction, fixed, and paraffin-embedded. The splenic sections (4 μm) from the individual mice were stained with anti-CD11c and intracellularly stained with anti-pIRF3 (S396), anti-pAKT (S473), or anti-pAKT (T308), followed by staining with fluorescent secondary antibody. The fluorescent signals were examined under a fluorescent microscope (Olympus 1000, Japan).

### Quantitative real-time PCR analysis

On days 24–26 post-immunization, five mice from each group were sacrificed, and their spleen and lumbar spinal cord tissues were dissected. Total RNA was extracted from the spinal cords or spleen using Trizol reagent (Life Technologies, Foster City, CA, USA) and reverse transcribed into cDNA using the HiFi-MMLV first-strand cDNA synthesis kit (CWbio, Beijing, China), according to the manufacturer’s protocol. The relative mRNA levels of IL-17A, RORγt, IFNγ, T-bet, and FOXP3 to the control β-actin were determined by qRT-PCR using RealSuper Mixture (Rox; CWbio) and specific primers (Table 1) in the Roche Light Cycler 480II system (Roche Diagnostics GmbH, Mannheim, Germany). The PCR reactions were performed in triplicate at 95 °C for 10 min and subjected to 40 cycles of 95 °C for 15 s and 60 °C for 60 s. The data were analyzed by the 2^−ΔΔCT^ method.

**Table 1:** The sequences of specific primers

Target gene	Forward	Reverse
IFN-γ	5′-CTGATCCTTTGGACCCTCTG-3′	5′-CAGCCATGAGGAAGAGCTG-3′
T-bet	5′-CAGTTCAACCAGCACCAGACAG-3′	5′-CCACCAAGACCACATCCACAAA-3′
IL-17	5′-TGCTACTGTTGATGTTGGGAC-3′	5′-AATGCCCTGGTTTTGGTTGAA-3′
RORγt	5′-GGTCCAGACAGCCACTGCATTC-3′	5′-GGTGCGCTGCCGTAGAAGGT-3′
FOXP3	5′-CTCTAGCAGTCCACTTCACCAA-3′	5′-CACCCACCCTCAATACCTCTCT-3′

### Enzyme-linked immunosorbent assay

The levels of IL-12, IL-23, and IL-6 in the supernatants of cultured BMDCs and IFN-γ, IL-17A, IL-10, IL-12, IL-23, and IL-6 in the supernatants of splenocytes were determined in triplicate by ELISA using specific kits, according to the manufacturer’s instructions (USCN LIFE).

### Statistical analysis

The experiments were performed in triplicate from three separate experiments. Data are expressed as the mean ± standard error of the mean (SEM). The difference between two groups was analyzed by the Mann-Whitney *U* test. Some data were first normalized, and the difference between two groups was analyzed by Student's *t* test. A *p* value of < .05 was considered statistically significant.

## Results

### ALX inhibits the LPS-induced proliferation and phenotypic maturation of BMDC

**Fig. 1:**
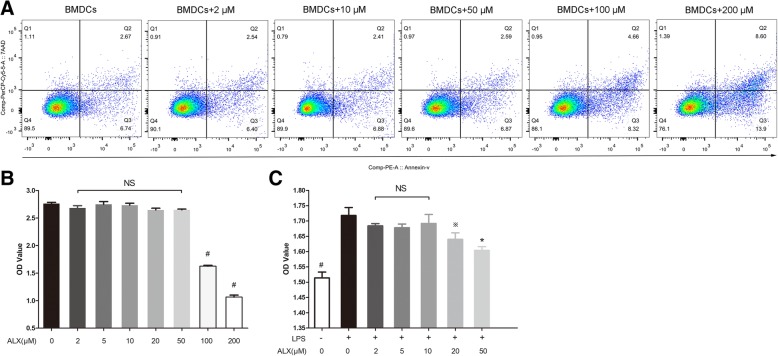
The effects of ALX on the survival and proliferation of BMDC. On day 7 of culture, BMDC (*n* = 6) were treated with or without ALX at the indicated dose for 12 h. Some DCs were further stimulated with LPS (1 μg/ml) for 48 h. The percentages of apoptotic in different groups of BMDCs were determined by flow cytometry analysis. The survival and LPS-stimulated proliferation of BMDCs were assessed by CCK-8 assays. **a** Flow cytometry analysis of apoptotic cells. **b** Survival of BMDCs from each group. **c** Proliferation of LPS-stimulated BMDCs from different groups. ^※^*p* < 0.05, *p < 0.01, ^#^*p* < 0.001, NS means no statistical difference vs. the cells treated with LPS alone

In this study, we first examined the effect of ALX treatment on the survival of BMDCs in vitro. Treatment with ALX between 2 and 50 μM did not affect the viability of BMDCs (Fig. 1a, b). However, treatment with ALX at a higher dose (100 or 200 μM) significantly reduced the viability and enhanced apoptosis of BMDCs (Fig. 1a, b). Accordingly, we choose doses ranging from 2 to 50 μM for the following experiment.

LPS can stimulate the proliferation and activation of BMDCs [40]. To investigate the effect of ALX on these processes, BMDCs were stimulated with, or without, LPS (1 μg/ml) in the absence or presence of various concentrations (2–50 μM) of ALX for 2 days. The proliferation of BMDCs was determined by CCK-8 analysis. Treatment with ALX at 20 or 50 μM, but not at a lower dose (2–10 μM), significantly inhibited the LPS-induced proliferation of BMDCs (Fig. 1c). Next, we chose two low doses of ALX (2 and 10 μM), which did not inhibit cell proliferation, for subsequent experiments to evaluate the impact of ALX on the maturation and immunoreactivity of BMDCs.

**Fig. 2:**
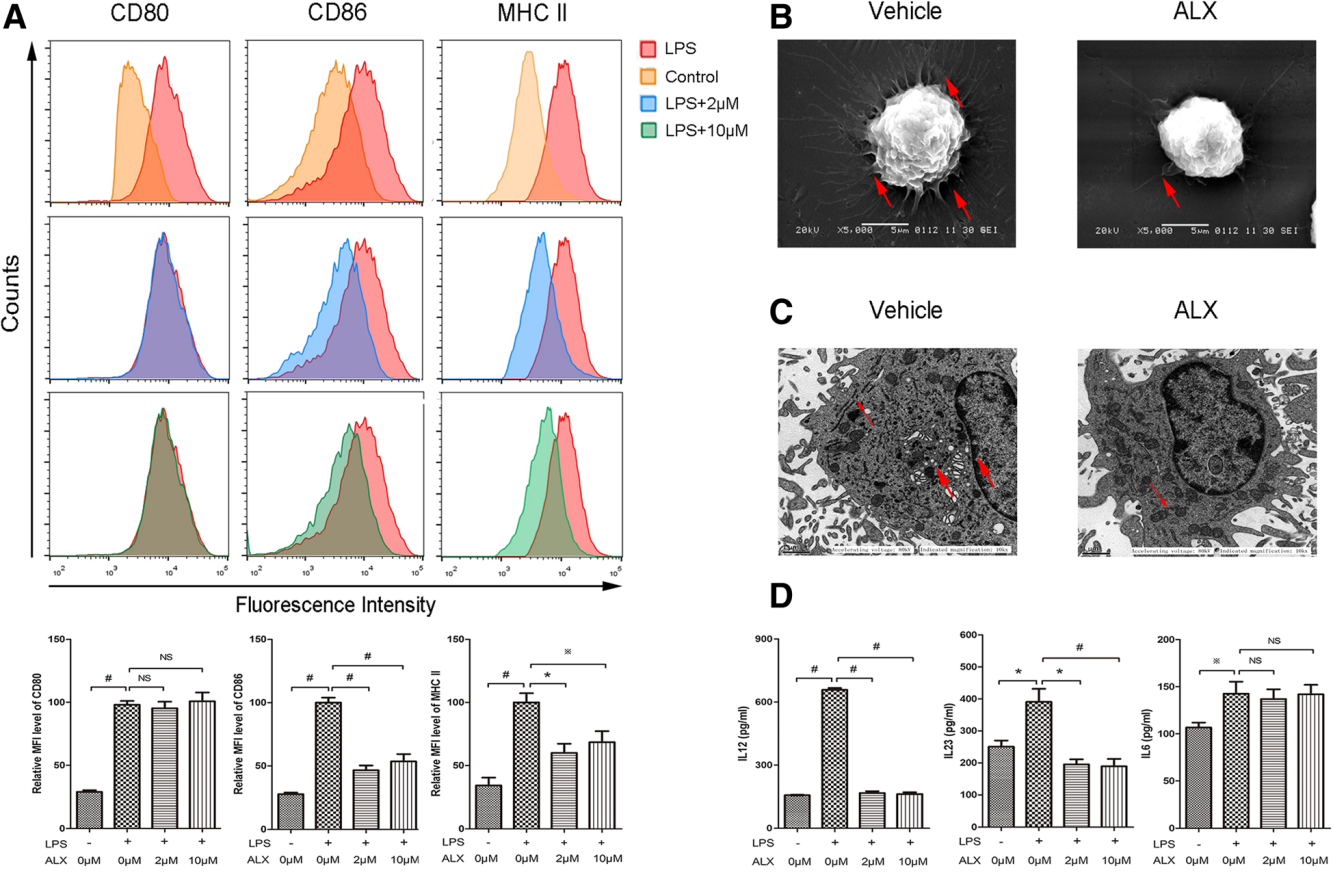
ALX inhibits LPS-induced maturation of BMDC. On day 8 of culture, BMDCs (*n* = 6) were stimulated with LPS (1 μg/ml) alone or together with ALX at 2 or 10 μM for 48 h. **a** Flow cytometric analysis of cell surface markers of CD80, CD86, and MHCII in BMDCs. **b** SEM analysis of BMDCs. The arrows indicate peripheral cellular protrusions. **c** TEM analysis of BMDCs. The thin arrow indicates well-developed rough endoplasmic reticulum, and the heavy arrow indicates Golgi apparatus. **d** ELISA analysis of IL-12, IL-23, and IL-6 levels in the supernatants of BMDCs. ^※^*p* < 0.05, ^*^p < 0.01, ^#^*p* < 0.001, NS means no statistical difference

To determine the impact of ALX on the maturation of DCs, mouse BMDCs from day 8 of culture were treated in triplicate with vehicle or LPS (1 μg/ml) in the presence or absence of ALX (2 and 10 μM) for 48 h. Given that mature DCs (mDCs) usually express high levels of MHCII and co-stimulatory factors (e.g., CD80 and CD86) on their surface, and display abundant long branched protrusions, and prominent Golgi apparatus (GA) and endoplasmic reticulum (ER), we characterized mDCs and immature DCs (imDCs) based on these indicators. Flow cytometric analysis showed that LPS substantially induce high levels (MFI) of CD80, CD86, and MHC II; co-treatment with ALX did not change CD80, but significantly reduced the levels (MFI) of CD86 and MHC II in LPS-activated BMDCs (Fig. 2a). SEM analysis displayed an abundance of long branched protrusions in LPS-treated BMDCs, but only a few of short branched protrusions in the ALX-treated cells (Fig. 2b). TEM analysis also revealed that the cytoplasm of LPS-treated BMDCs contained prominent GA and ER, but less rough ER and GA in the ALX-treated cells (Fig. 2c). As mDCs by LPS stimulation can produce high levels of pro-inflammatory cytokines, such as IL-12, IL-6, and IL-23, which are important for inducing Th1 and Th17 differentiation [43], we determined their levels in the supernatants of cultured BMDCs by ELISA. The results showed that ALX addition mitigated LPS-induced IL-12 and IL-23 production, but did not affect IL-6 production at the indicated doses (Fig. 2d). Collectively, these data demonstrated that treatment with ALX suppressed the LPS-induced maturation of BMDCs in vitro.

### ALX suppresses BMDC-mediated stimulation of antigen-specific T cell response

**Fig. 3:**
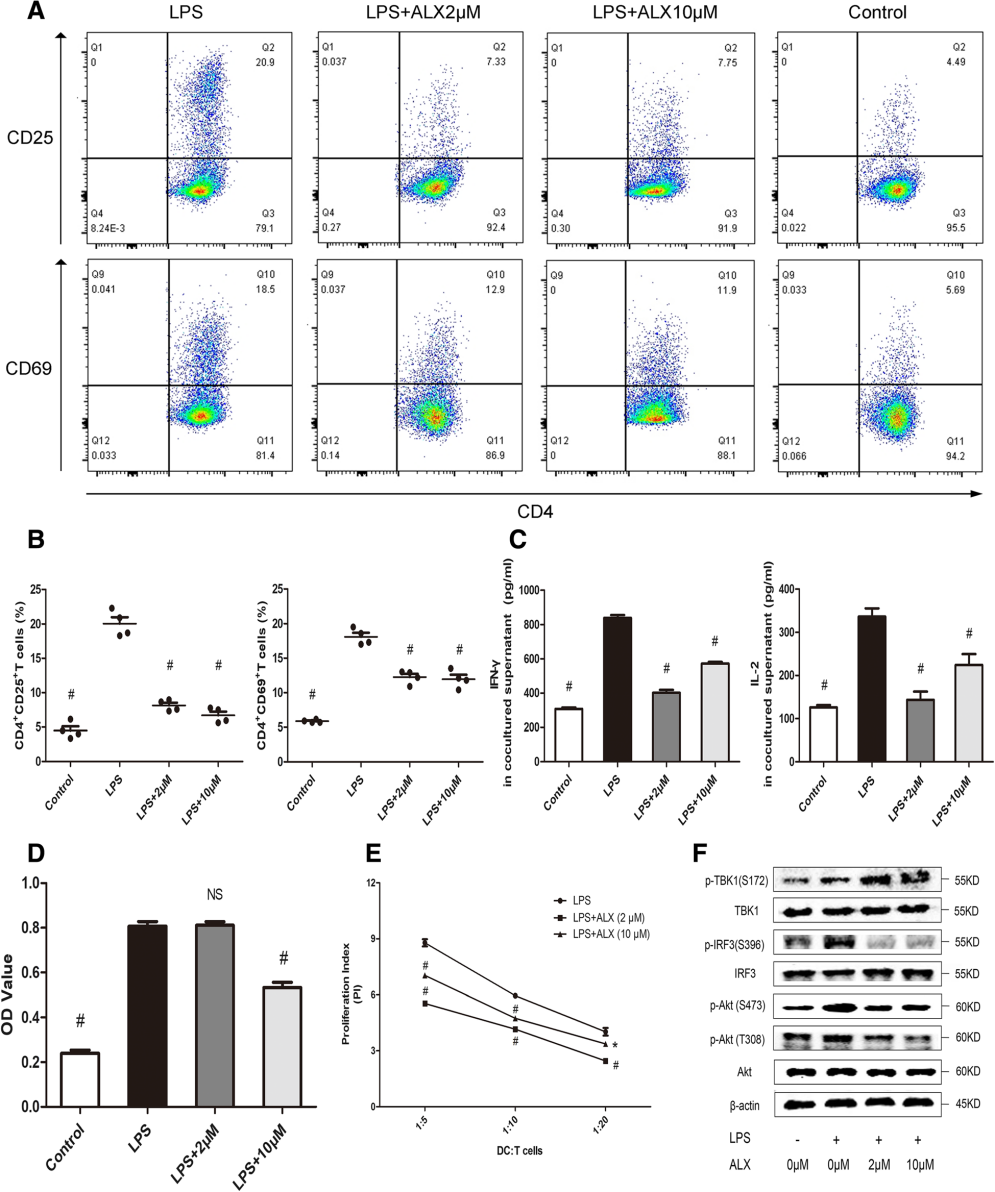
ALX impairs antigen-specific CD4+ T cell responses and allogeneic T cell proliferation. MOG-loaded BMDCs were stimulated with LPS (1 μg/ml) alone or together with the indicated doses of ALX for 48 h. Different groups of cells were cultured with splenic T cells at the indicated ratios for 3 days. The effect of ALX on TBK1, IRF3, and AKT expression and phosphorylation in the different groups of cells was determined by western blot assays. Similarly, splenic DCs from C57BL/6 mice were loaded with MOG antigen peptide and stimulated with LPS in the presence or absence of ALX at the indicated dose for 2 days. Some cells were pre-treated with mitomycin for 30 min. The different groups of DCs were co-cultured with splenic CD4^+^ T cells from MOG-immunized mice at 1:5 for 4 days. The frequency of CD4+CD25+ and CD4+CD69+ T cells was determined by flow cytometry, and the levels of IFNγ and IL-2 in the supernatants of co-cultured cells were measured by ELISA. The proliferation of T cells was determined by CCK-8 assay. **a** Flow cytometry analysis of CD4+CD25+ and CD4+CD69+ T cells. **b** Quantitative analysis of activated T cells. **c** The levels of IFN-γ and IL-2. **d** Proliferation of antigen-specific CD4+ T cells. **e** BMDC-stimulated allogeneic T cell proliferation. **f** Western blot analysis of TBK1, p-TBK1(S172), IRF3, p-IRF3(S396), AKT, and p-AKT (S473 and T308) expression following co-cultured with allogenic cells. ^※^*p* < 0.05, *p < 0.01, ^#^*p* < 0.001 the cells treated with LPS alone

Given that DCs are the cardinal stimulators of antigen-specific T cell responses, we next sought to determine if ALX could affect the DC-mediated antigen-specific T cell responses in vitro. BMDCs were pre-loaded with MOG peptide (10 μg/ml) and stimulated with LPS in the presence or absence of ALX (2 and 10 μM) for 2 days. Some DCs were pre-treated with mitomycin c for 30 min. The different groups of DCs were co-cultured with splenic CD4+ T cells from MOG peptide-immunized mice at a ratio of 1:5 for 4 days. Results revealed that co-culture with ALX-treated DCs significantly reduced the frequency of CD4+CD25+- or CD4+CD69+-activated T cells (Fig. 3a, b). Concomitantly, ALX treatment also significantly decreased the levels of IL-2 and IFNγ which correspond to the T cell activation [44, 45] in the supernatants of co-cultured cells (Fig. 3c). In addition, co-cultured with 10 μM, but not 2 μM ALX-treated DCs reduced the proliferation of antigen-specific CD4+ T cells (Fig. 3d). Thus, ALX inhibits the DC-stimulated MOG-specific T cell activation and proliferation in vitro.

### ALX suppresses BMDC-stimulated allogeneic T cell proliferation

Loosely adherent cells from day 8 of culture were used as immature BMDCs (imBMDCs) and 65% or more of non-adherent cells expressed CD11c^+^ [35]. The imBMDCs were stimulated with LPS to induce DC maturation in the presence or absence of ALX (2 or 10 μM) for 2 days. The BMDCs were purified by CD11c MicroBeads and treated with mitomycin C (proliferation blocker, 30 μg/ml) for 30 min. The BMDCs were co-cultured with allogeneic splenic CD4^+^ T cells purified the unmanipulated BALB/c mice at the indicated ratios for 48 h. The proliferation of CD4^+^ T cells was determined by CCK-8 assay. As shown in Fig. 3e, LPS-stimulated BMDCs promoted CD4^+^ T cell proliferation in a ratio-dependent manner. However, treatment with ALX at 2 μM, but not at 10 μM, significantly suppressed the BMDC-mediated allogeneic T cell proliferation.

### ALX inhibits the TBK1/AKT and TBK1/IRF3 signaling in BMDC

To explore the molecular mechanisms underlying the action of ALX in regulating the phenotypical and functional maturation of BMDCs, the phosphorylation of TBK1 and its associated proteins (IRF3 and AKT) in the different groups of BMDCs were determined by Western blot. As shown in Fig. 3f, although treatment with LPS did not change the expression of TBK1, IRF3, and AKT, it did increase their phosphorylation (p-AKT-T308, p-AKT-S473, p-IRF3-S396, p-TBK1-S172). Treatment with ALX further enhanced p-TBK1-S172 but mitigated the LPS-induced phosphorylation of AKT-T308, AKT-S473, and IRF3-S396.

### ALX treatment alleviates the severity of EAE in mice

**Figure 4:**
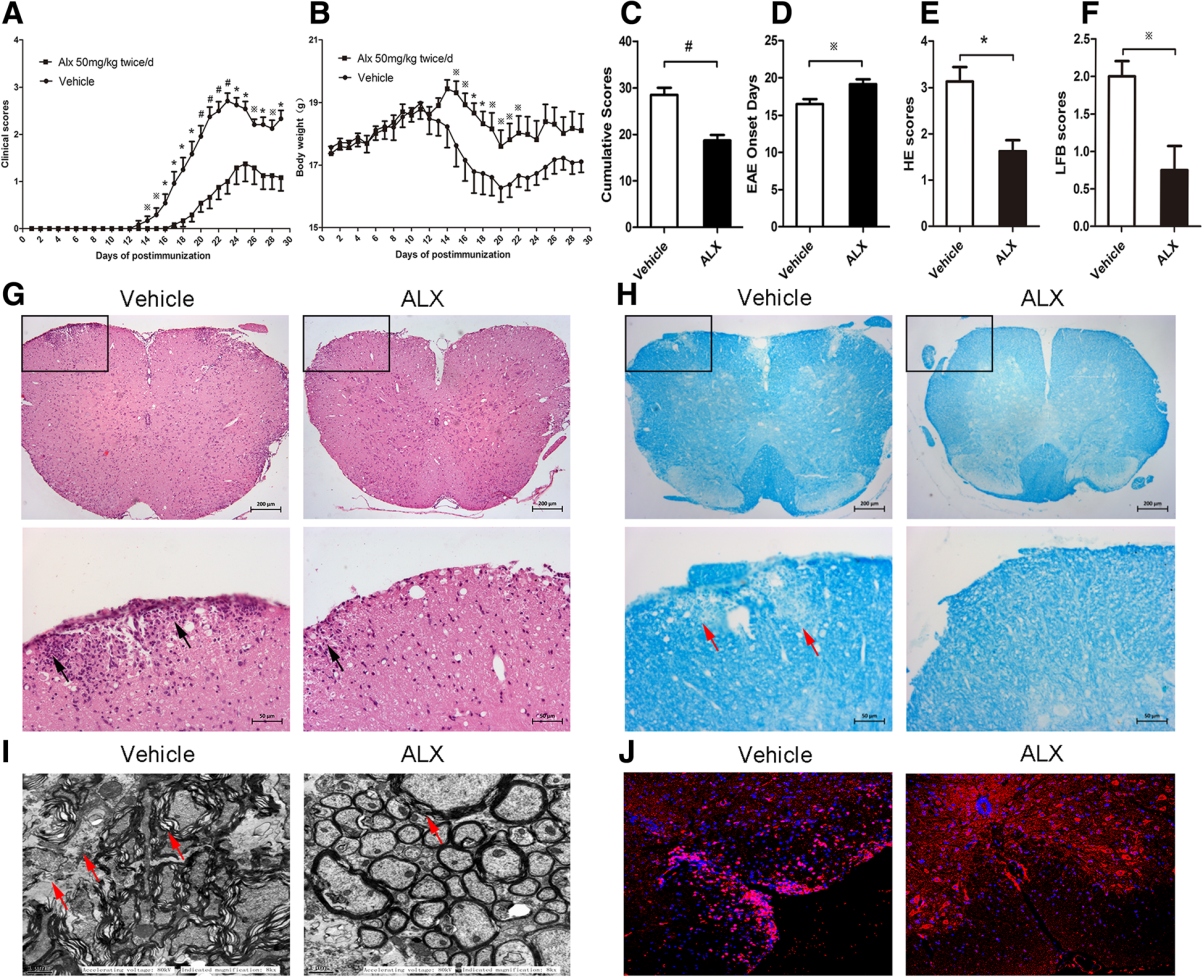
ALX treatment alleviates the severity of EAE in mice. C57BL/6 mice were injected with MOG35-55 in 50% CFA to induc e EAE and the mice were randomized and treated with vehicle or ALX (from day 0). We measured **a** the mean clinical scores of each group of mice; **b** the changes in their body weights; **c** the cumulative scores of disease severity; **d** the EAE onset days were calculated from the immunization day to the day of EAE clinical manifestation beginning; **e**, **f** the mean pathological scores for inflammation and demyelination in vehicle and ALX-treated mice were measured by semi-quantitative analysis; **g** the HE analysis of the spinal cord shows the infiltration of inflammatory cells in the white matter. The arrows indicate the inflammatory cells. **h** LFB analysis of the spinal cord shows the areas of intact myelin (blue) and demyelination (white). The arrows indicate the demyelination in the spinal cord. **i** Electron microscopic analysis of the lumbar enlargement. The arrows indicate the loose and loss of myelin in the spinal cord. **j** Immunofluorescent analysis of anti-F4/80 stained macrophages and microglia in the lumbar spinal cord tissue sections from the vehicle-treated and ALX-treated mice. Data are representative images or expressed as the means ± SEM of each group (*n* = 12). ^※^*p* < 0.05, *p < 0.01, ^#^*p* < 0.001

Next, we evaluated the potential therapeutic effect of ALX on the development and progression of EAE in mice. The dynamic development and progression of EAE in the different groups of mice were monitored for the disease scores (Fig. 4a), and their body weights were measured (Fig. 4b). ALX treatment significantly retarded the development and severity of EAE as measured by disease score; it also prevented weight loss. The cumulative disease scores in ALX-treated mice were significantly lower (Fig. 4c) and EAE onset later than in vehicle-treated mice (control mice) (Fig. 4d). Such data indicated that ALX treatment attenuated the development and progression of EAE in mice.

At the peak stage of EAE (day 24–26), spinal cord tissues were dissected and stained with H&E and LFB to determine the impact of ALX treatment on the severity of inflammation and demyelination. As shown in Fig. 4g, h, inflammation and demyelination were significantly reduced in the white matter of spinal cord from ALX-treated mice compared with control mice. Quantitative analysis showed that the inflammatory and LFB (demyelination) scores in the ALX-treated mice were significantly lower than control (Fig. 4e, f). Similar patterns of demyelination were observed in the spinal cord tissue sections by TEM (Fig. 4i). Immunofluorescent staining revealed that treatment with ALX decreased the anti-F4/80-stained macrophage and microglial signals in the lumbar spinal cord tissue sections (Fig. 4j). Taken together, our data demonstrated that ALX alleviated the severity of EAE in mice by reducing inflammatory infiltrates and demyelination in the spinal cord tissues.

### ALX treatment inhibits Th1 and Th17 responses in EAE mice

**Fig. 5:**
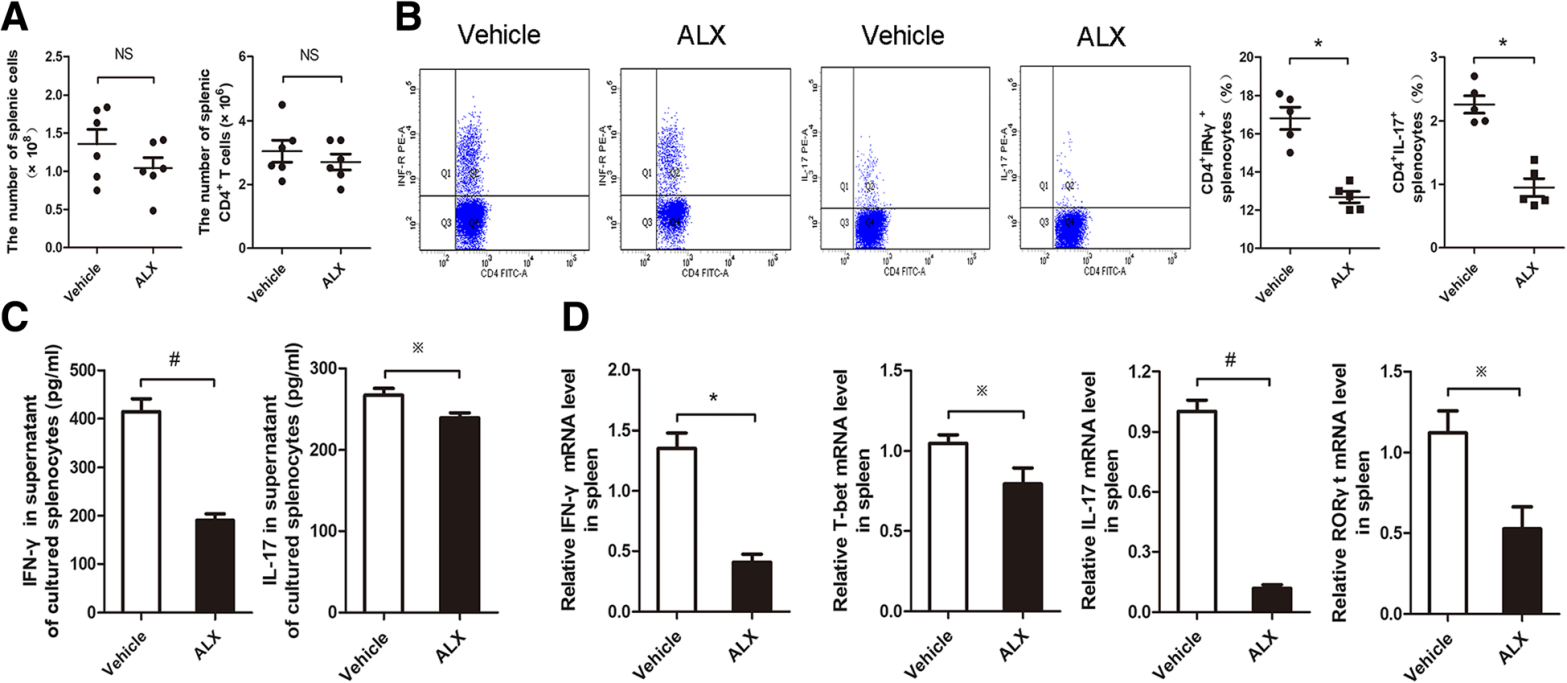
ALX treatment inhibits Th1 and Th17 responses in peripheral lymphoid organs of EAE mice. On day 25 post-induction, 5–6 mice in each group were sacrificed. The total numbers of splenic mononuclear cells and CD4+ T cells were calculated. Their splenic Th1 and Th17 cells were characterized by flow cytometry. The levels of splenic IFN-γ and IL-17 were determined by ELISA, and IFN-γ, IL-17, T-bet, and RORγt were determined by quantitative RT-PCR. Data are representative images or expressed as the means ± SEM of each group from three separate experiments. **a** Total number of splenic mononuclear cells and CD4+ T cells. **b** Flow cytometry analysis of the percentages of splenic Th1 and Th17 cells in the vehicle- and ALX-treated mice. **c** ELISA analysis of IFN-γ and IL-17 production in the supernatants of cultured splenocytes. **d** The relative levels of IFN-γ, T-bet, IL-17, and RORγt mRNA transcripts in the spleen of individual mice. ^※^*p* < 0.05, *p < 0.01, ^#^*p* < 0.001

Because inflammatory Th1 and Th17 cells are responsible for the pathogenesis of EAE, we further explored the effect of ALX on Th1 and Th17 responses in mice. Although there was no significant difference in total numbers of splenic mononuclear cells between these two groups of mice, the absolute numbers of splenic CD4+ T cells in ALX-treated mice decreased at the peak stage (Fig. 5a). In addition, the percentages of splenic Th1 and Th17 cells in ALX-treated EAE mice were significantly lower than in control mice (Fig. 5b). The levels of IFN-γ and IL-17A in the supernatant of cultured splenocytes from the ALX-treated mice were significantly lower than that from the untreated mice (Fig. 5c). Quantitative RT-PCR indicated that the relative levels of IFN-γ (Th1), T-bet (Th1), RORγt (Th17), and IL-17A (Th17) mRNA transcripts in the splenocytes of ALX-treated mice were significantly lower than in controls (Fig. 5d).

**Fig. 6:**
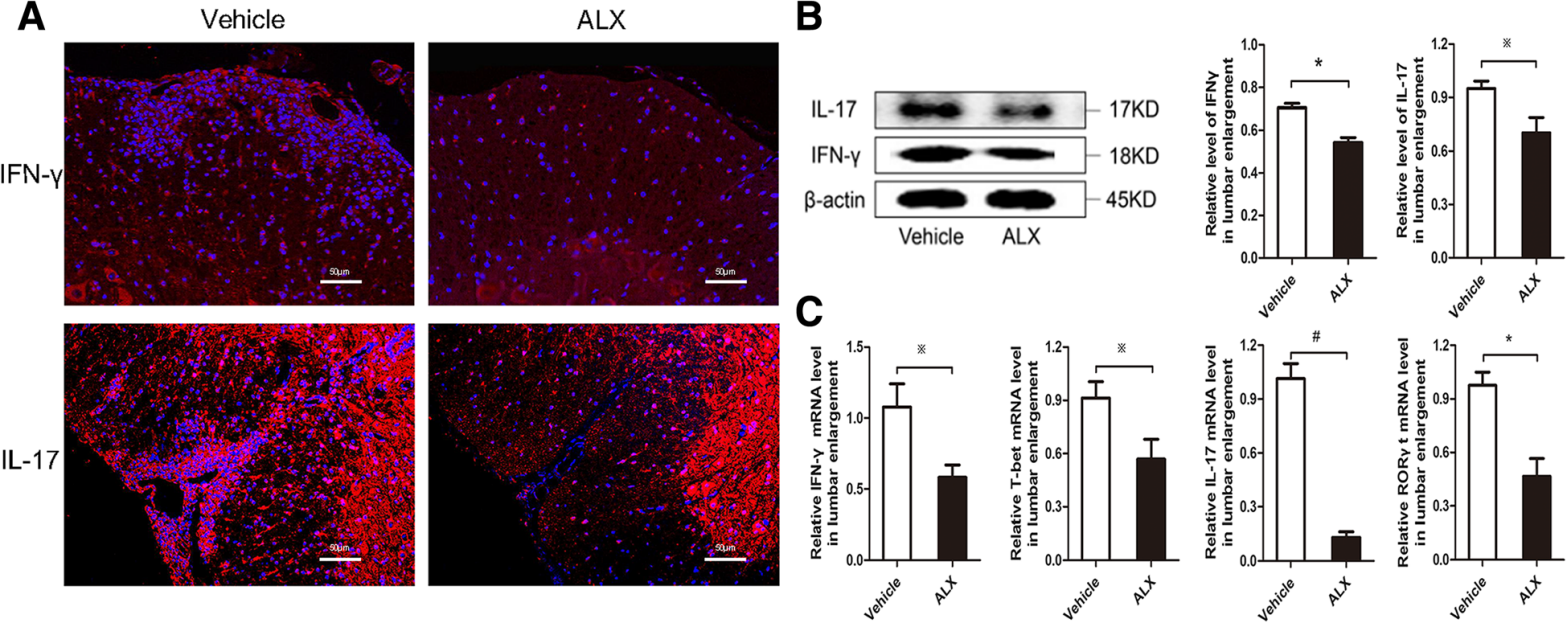
ALX treatment reduces the infiltration of Th1 and Th17 cells in the lumbar enlargement of EAE mice. On day 25 post-induction, some mice from each group were sacrificed, and their spinal cord tissues were characterized for the content of infiltrated Th1 and Th17 cells by immunofluorescence using anti-IL-17 and anti-IFN-γ antibodies. The relative levels of IL-17 and IFN-γ expression in the spinal cord tissues were determined by western blot and quantitative RT-PCR. Data are representative images or expressed as the means ± SEM of each group (*n* = 5) from three separate experiments. **a** Immunofluorescent analysis of Th1 and Th17 cells in the spinal lumbar enlargement. **b** Western blot analysis of IL-17 and IFN-γ expression in the lumbar enlargement. **c** The relative levels of IFN-γ, T-bet, IL-17, and RORγt mRNA transcripts in the lumbar enlargement of individual mice. ^※^*p* < 0.05, *p < 0.01, ^#^*p* < 0.001

Pathogenic Th1 and Th17 cells can migrate and induce inflammation and demyelination in the spinal cord tissue. To understand the effect of ALX on this process, we further determined the presence and function of Th1 and Th17 cells in the lumbar regions of the spinal cord by immunofluorescence. As shown in Fig. 6a, there were lesser IFN-γ^+^ and IL-17A^+^ cells from ALX-treated mice than from control mice. Western blot and RT-PCR revealed that the expression of IFN-γ and IL-17A protein and IFN-γ, T-bet, RORγt, and IL-17A mRNAs in the lumbar regions from the ALX-treated mice were significantly reduced, as compared with the controls (Fig. 6b, c). These data demonstrated that treatment with ALX inhibited Th1 and Th17 responses in both the peripheral lymphoid organ (spleen) and spinal cord tissues of EAE mice.

### ALX enhances regulatory T cell response

**Fig. 7:**
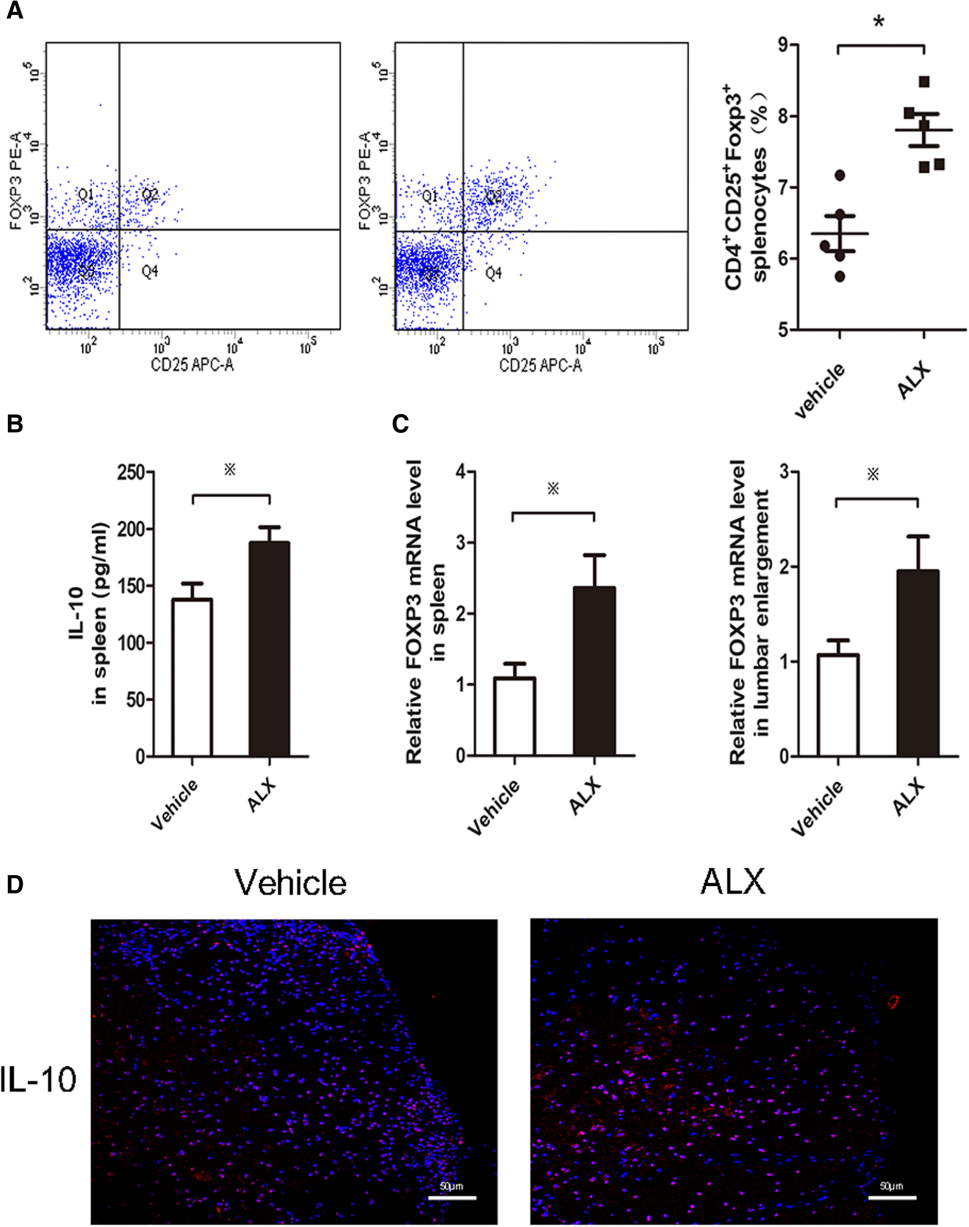
ALX treatment increases the infiltration of FOXP3^+^ Tregs and IL-10 secretion in the spleen and lumbar enlargement of EAE mice. On day 25 post-induction, five mice from each group were sacrificed, and their splenic CD4^+^CD25^+^FOXP3^+^Tregs were characterized by flow cytometry. The levels of splenic IL-10 were determined by ELISA and FOXP3 mRNA transcripts in the spleen, and lumbar enlargement was determined by quantitative RT-PCR. Their spinal cord tissues were characterized for the content of infiltrated Tr1 cells by immunofluorescence using anti-IL-10 antibody. Data are representative images or expressed as the means ± SEM of each group (*n* = 5) from three separate experiments. **a** Flow cytometry analysis of CD4^+^CD25^+^FOXP3^+^ Tregs. **b** ELISA analysis of IL-10 production in the supernatants of cultured splenocytes. **c** The relative levels of FOXP3 mRNA transcripts in the spleen and lumbar enlargement of individual mice. **d** Immunofluorescent analysis of Tr1 cells in the spinal lumbar enlargement

Since the impaired Treg responses are involved in the pathogenesis of EAE and Tregs can limit EAE progression [46], we further examined whether ALX could influence the response of Tregs in the CNS and spleen. The percentages of splenic CD4^+^CD25^+^FOXP3^+^ Tregs in ALX-treated EAE mice were higher than in control mice (Fig. 7a). The levels of IL-10 in the supernatants of cultured splenocytes from the ALX-treated mice increased, compared with that from the control mice (Fig. 7b). Quantitative RT-PCR indicated that the relative levels of FOXP3 mRNA transcripts in the splenocytes and lumbar regions of ALX-treated mice were significantly higher than in vehicle controls (Fig. 7c). Furthermore, there were more IL-10^+^ cells in the lumbar regions of the spinal cord from ALX-treated mice than from control mice (Fig. 7d). These data indicated that ALX upregulated FOXP3^+^ Treg responses and their IL-10 secretion to protect mice from EAE.

### ALX suppresses DC maturation in EAE mice

**Fig. 8:**
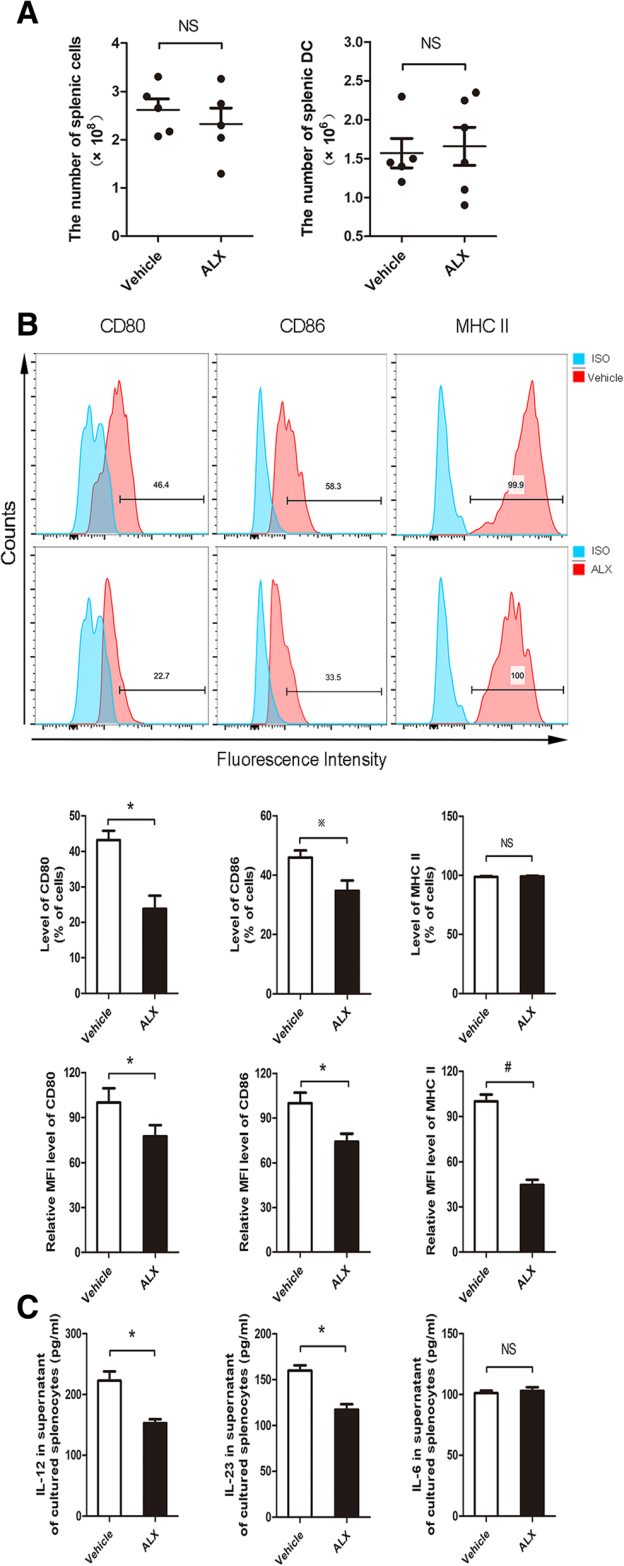
ALX suppresses DC maturation in EAE mice. On day 15 post-induction, five mice from each group were sacrificed. The total number of splenic mononuclear cells and DCs were calculated. Splenic mononuclear cells were stained with anti-CD80, anti-CD86, and anti-MHC II. The levels of CD80, CD86, and MHC II expression on DCs indicate the degrees of DC maturation. The levels of IL12, IL-23, and IL-6 in cultured cell supernatants of individual mice were determined by ELISA. Data are representative images or expressed as the mean ± SEM of each group (*n* = 5) from three separate experiments. **a** Total number of splenic mononuclear cells and DCs. **b** Flow cytometry analysis of splenic DC maturation. The percentage of splenic CD80+CD11c+, CD86+CD11c+, and MHC II+CD11c+ cells in individual mice from different group. The MFI of CD80, CD86, and MHC II on DCs. **c** The expression levels of IL-12, IL-23, and IL-6 in supernatants of cultured splenocytes.^※^*p* < .05, *p < .01, ^#^*p* < 0.001, NS means no statistical difference

**Fig. 9:**
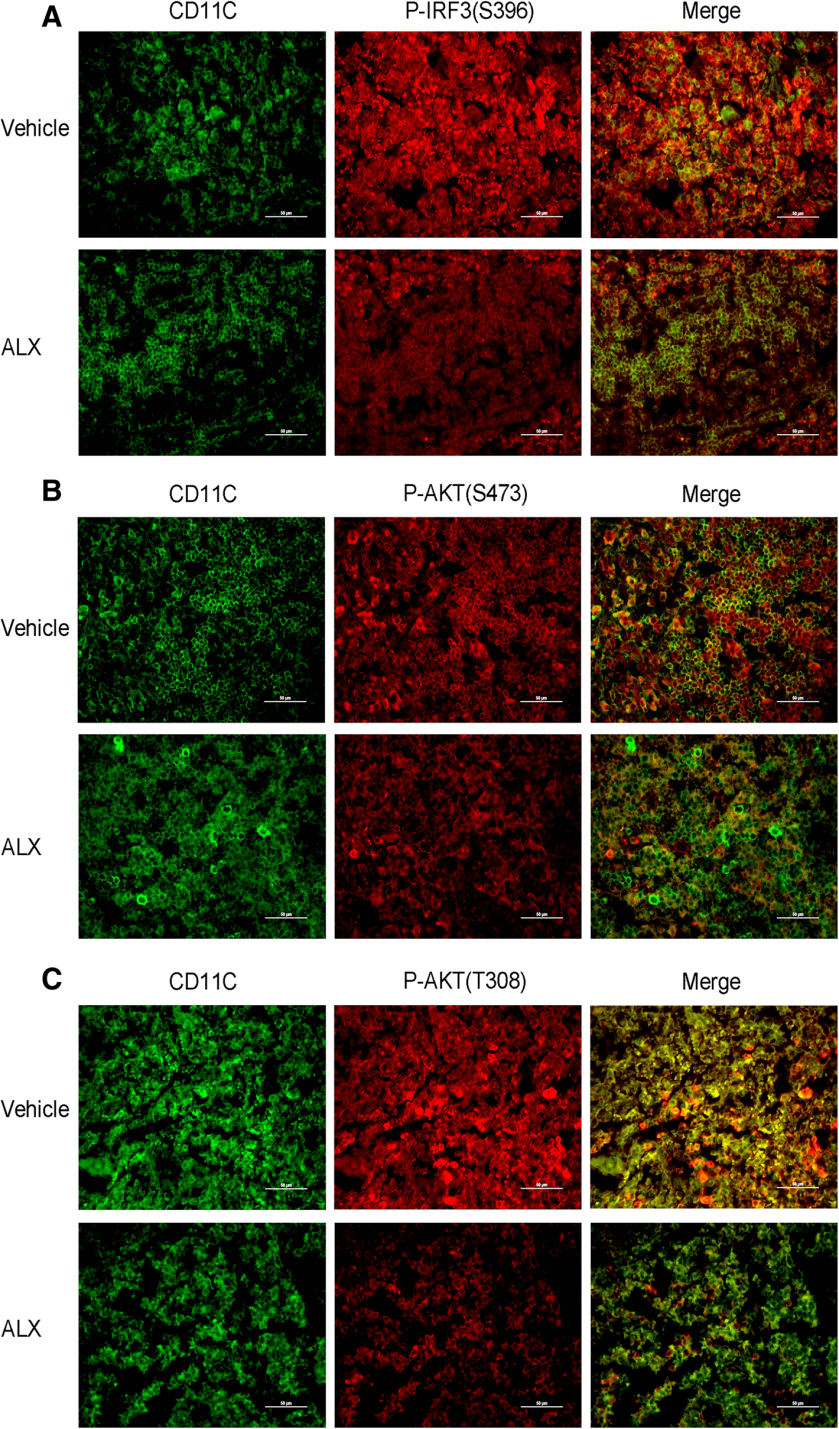
ALX minimizes the TBK1-AKT and TBK1-IRF3 signaling in splenic DCs. At the peak stage of EAE, some mice from each group were sacrificed, and their splenic tissue sections were stained with anti-CD11c (green) and anti-p-IRF3 (red) or anti-p-AKT (red) antibodies. Representative images from each group are shown. **a** ALX reduces the p-IRF3 signals in the splenic tissues. **b**, **c** ALX mitigates the AKT activation in the splenic tissues

Given that DCs are crucial for inducing antigen-specific functional T cell differentiation in vivo, we further investigated whether ALX could modulate the numbers and maturation of splenic DCs in EAE mice. Splenic mononuclear cells were isolated from ALX-treated and control EAE mice on day 15 post-induction (early stage of EAE). The total numbers of splenic mononuclear cells and DCs were calculated. The frequency of CD80^+^, CD86^+^, and MHCII^+^ cells and the expression (MFI) of CD80, CD86, and MHC II in different groups of DCs (CD11C^+^ cells) were characterized by flow cytometry. In the early stage of EAE, there was no significant difference in the total numbers of splenic mononuclear cells and DCs between the ALX-treated and control groups of mice (Fig. 8a). In comparison with the control, ALX significantly decreased the frequency of CD80^+^ and CD86^+^, but not MHC II^+^ cells, and potently reduced the expression (MFI) of CD80, CD86, and MHC II in CD11c^+^ DCs (Fig. 8b). Because activated DCs can secrete pro-inflammatory cytokines to enhance T cell autoimmunity [47], we determined the levels of splenic IL-12, IL-6, and IL-23 in the vehicle- and ALX-treated mice by ELISA. The levels of IL-12 and IL-23, but not IL-6, in the ALX-treated mice were significantly lower than in control mice (Fig. 8c). We next explored the possible signaling pathway affecting the maturation of DC. Immunofluorescent analysis revealed that fluorescence intensity of p-IRF3(S396), p-AKT(S473), and pAKT(T308) in CD11c^+^ DC cells in the spleens of ALX-treated mice were lower than those in control mice (Fig. 9a–c). Collectively, these data indicated that ALX, an inhibitor of TBK1, attenuated the maturation of DCs along with inhibiting the TBK1/AKT and TBK1/IRF3 signaling in splenic DCs of EAE mice.

## Discussion

A previous study has shown that conditional knockout of TBK1 in T cells mitigates EAE development via impeding the effector T cells egressing from peripheral lymphoid organs [28]. In this study, we found that ALX prevented maturation of DCs, decreased frequency of pathogenic Th1 and Th17 cells, and increased proportions of Treg cells, and attenuated EAE progression in mice. Our findings provide new pharmacological insight into the biological function and mechanism of ALX as a promising drug for the intervention of MS.

DCs are the predominant antigen presenting cells, and their maturation is important for antigen presentation, T cell activation, proliferation, and functional differentiation (e.g., Th1, Th17, and Treg cells), the balance of which is crucial for the pathogenesis of EAE and MS [48]. The mature DC preferentially polarize T cells into Th1 and Th17 lineage [49]. It is critical to induce robust inflammatory and immune responses to eliminate foreign intruders (e.g., pathogens and microbes), but can be harmful in acute progressive stage of certain inflammatory or autoimmune diseases, such as MS. Immature and semi-mature DCs with low surface expression of co-stimulatory molecules and MHC class II are not only crucial to inducing Tregs for clearing out exogenous microbes, potentially leading to chronic inflammation, but are also important for maintaining immune homeostasis and tolerance [20, 50].

The conditional knockout of TBK1 in DCs or T cells led to a completely distinct outcome for EAE induction. The ablation of TBK1 in DCs sensitizes EAE induction, while the deletion of TBK1 in T cells resists EAE development [28, 51]. Although administration of ALX can hinder EAE progression, the mechanisms underlying the action of ALX for the beneficial effect remain to be fully elucidated. Our in vitro data indicated that treatment with ALX significantly impaired the phenotypical maturation of LPS-stimulated BMDCs by reduced MHCII and co-stimulatory molecule (CD86) expression and morphological changes (shortened branched protrusions and less prominent GA and ER). ALX treatment also decreased the secretion of IL-12 (Th1 differentiation cytokine) and IL-23 (Th17 differentiation cytokine). In addition, ALX inhibited functional maturation (immunoreactivity) of BMDCs. ALX-treated BMDC displayed a diminished ability to stimulate allogeneic T cell proliferation and antigen-specific T cell responses (proliferation and activation). Our in vivo data also revealed that ALX reduced the frequency and expression of co-stimulatory molecules (CD80^+^ and CD86^+^) in CD11c^+^ splenocytes, but did not affect the total number of DCs. All these results supported the ALX inhibited activation and maturation of LPS-induced DC and splenic DC from EAE mice.

As expected, we found that ALX attenuated EAE development with delayed onset and decreased the severity of the disease. In the lumbar spinal cords, ALX decreased inflammatory infiltrates and demyelination characterized by fewer IFN-γ^+^ and IL-17A^+^ T cells, and reduced IFN-γ, T-bet, IL-17A, and RORγt expression compared with vehicle-treated EAE mice. Although there is no differences in total number of splenic mononuclear cells and splenic CD4^+^ cells, the percentages of CD4^+^IFN-γ^+^(Th1) and CD4^+^IL17A^+^(Th17) effector T cells also decreased in the ALX-treated mice at the peak stage of EAE induction, coupled with reduced levels of IFN-γ and IL-17A production in the supernatant, and lowered mRNA levels of IFN-γ, T-bet, IL-17A, and RORγt in spleen tissues. These indicated that ALX inhibited pathogenic Th1 and Th17 responses not only in the CNS but also in the spleen.

Tregs in MS was initially documented by studies in EAE because adoptive transfer of Tregs ameliorated the disease, while the depletion of Tregs worsened the disease [52, 53]. The functional impairments of Tregs and IL-10-producing Tr1 cells are involved in MS and EAE development [54, 55]. Our data revealed that the FOXP3 mRNA levels increased in both the CNS and spleen of ALX-treated EAE mice, coupled with enhanced expression of IL-10. The flow cytometric analysis displayed the increased frequency of FOXP3^+^CD25^+^ Tregs in splenocytes of ALX-treated EAE mice, further indicating the involvement of Tregs in ALX-mediated EAE attenuation.

IRF-3 expression is regulated by TBK1/IKKε in innate immune responses [30, 56]. Downregulation of IRF-3 in DCs by siRNA or drugs inhibited their phenotypic and functional maturation [31, 32], and irf3^−/−^ mice developed less severe EAE through reduced Th17 response [57]. Therefore, ALX may impede DC maturation and subsequent pro-inflammatory cytokine production, at least partially, through inhibiting the TBK1/IRF3 signaling, and further diminish effector T cell responses and EAE progression. TBK1/IKKε can also activate the AKT signaling by phosphorylating AKT at Ser473 and Thr308 [58, 59]; knockdown of TBK1 by siRNA inhibits AKT activation in DCs [60]. Activation of the AKT signaling can promote DC maturation by enhancing Bcl-2 expression and NF-kB activation [61, 62], and is necessary for hexokinase II (HK-II) activation and subsequent glycolysis. The increased glycolysis plays a vital role in DC activation and facilitates the synthesis of the ER and Golgi membrane [33]. In contrast, inhibition of AKT reduces glycolytic activity, leading to impairment of DC maturation [63, 64]. In line with these reports, our results displayed that ALX markedly inhibited IRF-3 and AKT phosphorylation, which in turn may impair DC maturation, reducing pathogenic Th1 and Th17 responses in the EAE mice.

Currently, there are several other therapeutic drugs available for MS treatment or in clinical trials, including IFN-β, mitoxantrone, glatiramer acetate, and fingolimod [65, 66]. However, the therapeutic efficacy of these reagents is limited, and some of which even have palpable side-effects [67]. ALX has been used for more than 25 years in the clinic. To the best of our knowledge, this was the first study to elucidate that ALX mitigated EAE development through inhibiting DC maturation and subsequent pathogenic Th1 and Th17 responses while increasing regulatory T cell responses. Thus, our findings may aid in exploring new therapies for the intervention of MS.

## Conclusions

In summary, our data indicated that ALX inhibited the development and progression of EAE and mitigated inflammation and demyelination, accompanied by reducing antigen-specific Th1 and Th17 response while boosting Treg responses by inhibiting DC maturation through attenuating the TBK1/Akt and TBK1/IRF3 signaling in BMDC and splenic DC. Therefore, ALX may attenuate EAE by inhibiting dendritic cell maturation through inhibiting TBK1 and reprogramming effector and regulatory T cell responses.

## Abbreviations

ALX: Amlexanox
APCs: Antigen presenting cells
BMDCs: Bone marrow-derived dendritic cells
CNS: Central nervous system
DCs: Dendritic cells
EAE: Experimental autoimmune encephalomyelitis
ER: Endoplasmic reticulum
GA: Golgi apparatus
IKKε: IkB kinase ε
imBMDCs: Immature BMDCs
imDCs: Immature DCs
mDCs: Mature DCs
MLR: Mixed lymphocyte reaction
MS: Multiple sclerosis
SEM: Scanning electron microscopy
TBK1: TANK-binding kinase 1
TEM: Transmission electron microscopy
Tr1: Regulatory type 1
Tregs: Regulatory T cells
